# Effects of a combination of non-pharmaceutical psychological interventions on dental anxiety

**Published:** 2017-09-29

**Authors:** Choon Yoong Wong, Coumaravelou Saravanan, Ammar Musawi, Shou Wan Gan

**Affiliations:** ^1^School of Dentistry, International Medical University, Kuala Lumpur, Malaysia; ^2^College of Medicine, University of Sharjah, Sharjah, United Arab Emirates; ^3^Restorative Dentistry Faculty, AT Still University, Kirksville, United States; ^4^School of Dentistry, International Medical University, Kuala Lumpur, Malaysia

**Keywords:** Dental Anxiety Scale-Revised (DAS-R), Dental Concern Assessment (DC A), psychological intervention, anxiety reduction

## Abstract

**Background:**

Dental anxiety is a common problem associated with poorer oral health. Managing anxiety is key to improving oral health of patients with dental anxiety. The present pilot study therefore investigated dental anxiety prevalence among patients visiting a university dental clinic. We further examined the effect of combined psychological interventions on anxiety or concern towards dental treatment procedures before treatment, after treatment, and at follow-up.

**Methods:**

In this prospective pilot study, patients seeking restorative treatment were screened for dental anxiety and dental concern about treatment using the Dental Anxiety Scale-Revised (DAS-R) and Dental Concern Assessment (DCA) questionnaires. Participants with a DAS-R score of 9 or above were randomly assigned to an experimental or control group. The patients in the experimental group received two psycho-logical interventions (psychoeducation and progressive muscular relaxation) prior to dental treatment. Dur-ing treatment, patients received another psychological intervention (music distraction). No psychological interventions were given to control patients. DAS-R and DCA scores were used to assess dental anxiety and concern, respectively, before treatment, after treatment, and at follow-up. Nonparametric tests were used for intergroup and intragroup statistical analyses.

**Results:**

Out of 64 patients surveyed, 33 (51.6%) had experienced dental anxiety. Of those, 2 were exclud-ed, and 31 patients with a mean ± SD age of 41.2 ± 15.9 y completed the study. No intergroup differences in dental anxiety were found in terms of pretreatment, posttreatment, and follow-up treatment. The mean rank value of the dental anxiety score was less in the experimental group (13.53) than the control group (18.31), albeit not significant. More specifically, differences (Kruskal-Wallis %2 = 14.82, *P* = 0.001, effect size = 0.33) were found in the experimental group for pretreatment, posttreatment, and follow-up treatment levels of dental anxiety for extraction (P = 0.01), injection (P = 0.02), and sound/vibration of the drill (P = 0.00). No significant intragroup differences between pretreatment, posttreatment, and follow-up treatment were found in the control group.

**Conclusions:**

The combined brief psychological interventions reduced dental anxiety.

**Relevance for patients:**

The psychological interventions of the present study could be applied right before or during dental treatment to reduce the dental anxiety of patients. However, additional research involving larger groups is needed to replicate the results of this pilot study.

## 1. Introduction

Dental anxiety is defined as anxiety associated with an an-ticipated encounter with a feared stimulus specific to an oral health care situation [1]. The *Diagnostic and Statistical Manu-al of Mental Disorders - IV* [2] describes dental phobia as “a marked and persistent fear of clearly discernible, circum-scribed objects or situations” and, thus, is placed in the cate-gory of specific phobias. In a 1998 United Kingdom dental health survey, 49% of the population was anxious about visit-ing the dentist [3]. In a Malaysian study, Abdul Jalil et al. [4] reported that 92.1% of adult patients attending a dental clinic at a university experienced dental anxiety.

The development of dental anxiety has been associated with a traumatic experience from previous dental visits [5]. People who lack oral health awareness are more likely to be afraid of visiting a dentist [6]. Wardle [7] found that the most powerful anxiety-provoking stimulus for many patients was dental in-jection. Because patients with dental anxiety tend to avoid necessary dental treatment and frequently cancel their sched-uled appointments, they are often difficult to treat and require more chair time once they are in the dental chair [8]. Misdiag-nosis of a dental condition may even occur because anxious patients are regarded as a major source of professional stress to dentists [9]. Avoidance of dental treatment due to anxiety is common and appears to be strongly associated with deteriora-tion of oral health [10]. These factors may also be related be-cause embarrassment may be another problem experienced by anxious patients who have not been to a dentist in a long time. Taken together, this could lead to a vicious cycle of cumulative anxiety and increasing avoidance [11].

Managing anxiety is one of the means to improve the oral health of patients with dental anxiety. Psychological and pha-rmacological methods can be used to treat dental anxiety [12]. Psychological treatments include psychological education, relaxation techniques, distraction strategies, hypnosis, behav-ioral management, and cognitive therapy [13-15]. Pharmaco-logical treatments involving the use of nitrous oxide sedation, intravenous conscious sedation, benzodiazepines, and general anesthesia have been effective in reducing dental anxiety [16,17]. Using a multifaceted approach rather than relying on one treatment method improves the likelihood of success in reducing dental anxiety. However, most psychological treat-ments require more time, while short-term treatments like medication may lead to increased patient risk from potential drug interactions [16]. Thus, there is a need for brief psycho-logical treatments that can be widely applied.

The present study assessed the prevalence of dental anxiety and concern among patients at a university dental clinic in Malaysia. We further determined the effect of a combination of psychological interventions (psychoeducation, progressive muscular relaxation, and music distraction) on dental anxiety and concern before, during, and after treatment. Intergroup and intragroup analyses were performed on the basis of the Dental Anxiety Scale-Revised (DAS-R) and the Dental Concern As-sessment (DCA) questionnaire scores. We hypothesized that the combined psychological interventions would result in re-duced dental anxiety and concern. The interventions as regards pretreatment, post treatment and follow-up treatment scores.

## 2. Methods

### 2.1. Patients

Ethics and study approval were acquired from the universi-ty’s institutional review board. Study participants were re-cruited prospectively and comprised patients who attended the Oral Health Center of the International Medical University in Malaysia. Based on a sample size calculation with 95% confi-dence and 5% margin of error, the present study needed a minimum of 64 patients attending the oral health center to be enrolled in the study. This corresponded to an inclusion period of 3 months based on historical patient visits at our clinic.

**Figure 1.**
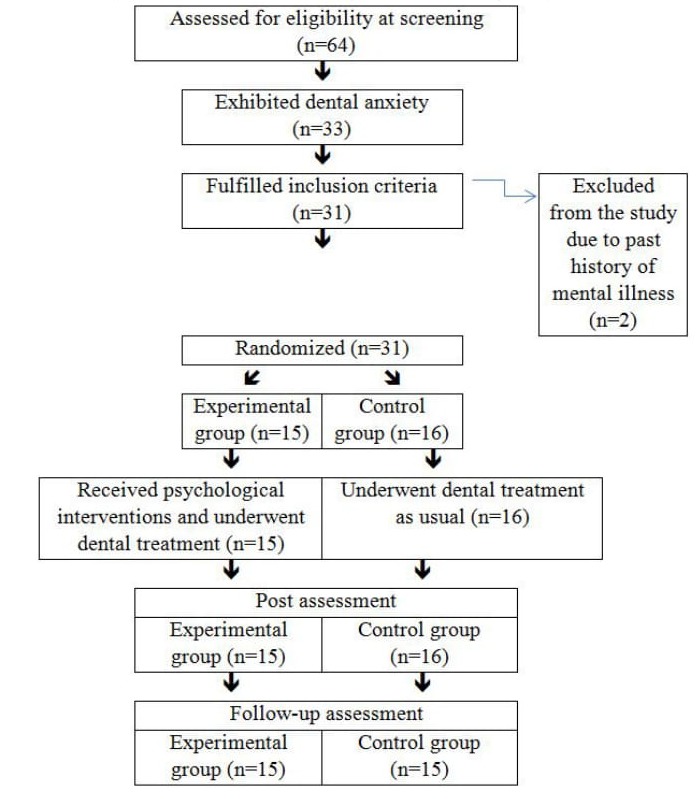
Summary of the experimental design and cohort size.

The experimental design up is provided in Figure 1 The pa-tients were informed about the study and consent was obtained from the patients before inclusion in the study. Patients who agreed to participate in the study were asked to fill in a demo-graphic information form, the DAS-R questionnaire, and the DCA questionnaire. A total of 31 patients (mean ± SD age of 41.2 ± 15.9 y) were randomly divided into an experimental group (N =15) and a control group (N =16) by using soft-ware-generated random numbers (SPSS, version 18.0, Chicago, IL). The participants were not aware of which group they had been assigned to until the experimental group received the intervention. The control group remained blinded to the allo-cation schedule throughout the entire experiment

Patients who have dental anxiety (DAS-R score of ^ 9 and DCA score of ^ 2) were included in this study. Participants who scored 4-8 points on the DAS-R scale and 1 on the DCA scale were excluded because these scores indicate no-to-low anxiety towards dental treatment. Moreover, patients with a history of mental illness or who were taking anxiolytic drugs were excluded from the study to ensure that the effect of the psychological interventions was not influenced by the use of pharmacological agents. A history of mental illness and the use of anxiolytic drugs were derived from the demographic infor-mation form.

### 2.2. Questionnaires

After providing informed consent, the participants com-pleted the demographic information form as well as the DAS-R and DCA questionnaires. The demographic form que-ried gender, age, educational status, and past and current men-tal illnesses. Only gender, age, and educational status were statistically analyzed.

The DAS-R was used to measure dental anxiety on the basis of a 5-point Likert scale. Scores range from 4 to 20 points. A score of 4-8 points is defined as no dental anxiety, 9-12 points as moderate anxiety, 13-14 points as high anxiety, and 15-20 points as severe anxiety. The DAS-R has been widely used by many researchers to measure dental anxiety [1] and has a Cronbach a-coefficient of 0.82, indicating adequate reliability [18,19].

The DCA [20] was employed to identify factors causing dental anxiety. The questionnaire consists of 26 items and is scored using a 4-point Likert scale. Participants who scored ^ 2 were considered to experience moderate-to-severe anxiety towards the dental treatment.

Each participant completed the DAC-R and DCA question-naire before and immediately after the restorative treatment. The same tests were used when the participants returned 2 weeks later for their treatment follow-up. All pretreatment, posttreatment, and follow-up treatment assessments were ad-ministered, monitored, and scored by researchers who were not involved in the dental procedures.

### 2.3. Psychological interventions

The researcher who administered the psychological inter-ventions was taught about psychological techniques as a part of his dental curriculum and also received training from a psychologist on how to use these psychological techniques on the patients.

#### 2.3.1. Experimental group

A combination of psychological interventions was used in the experimental group in the present study to reduce dental anxiety, specifically psychological education, progressive muscular relaxation, and music distraction. For psychological education, participants were educated verbally by the re-searcher about symptoms of dental anxiety, prevalence of den-tal anxiety, causes and effects of dental anxiety, available treatments for dental anxiety, and the rationale for dental anxi-ety treatments [21]. For progressive muscular relaxation, the participants were instructed to contract and release different muscle groups of the body. This technique was developed by Edmund Jacobson based on the theory that relaxation of mus-cles will lead to relaxation of the mind because "an anxious emotional state fails to exist in the presence of complete relax-ation of peripheral parts [22]." Instructions were played through a pair of headphones, and demonstrations were pro-vided by the researcher when required. Progressive muscular relaxation has been shown to be effective in reducing dental anxiety [23]. The last intervention, music distraction, was pro-vided when dental treatment commenced. All participants of the experimental group listened to a specific music album (Pravin Mani’s *Music for De-Stress & Relaxation)* through headphones during their dental treatments. The positive effects of music distraction are believed to arise from a combination of relaxation and distraction, minimizing the anxiety and re-ducing the pain experienced [24]. By allowing the participants to manipulate the music volume, a degree of control over their sensory experience was implemented while simultaneously masking the fear-enhancing noises from the dental drill [25]. Music distraction has been used successfully among dental patients and in other medical settings such as oncology [26].

#### 2.3.2. Control group

Control group participants received the usual treatment for their dental anxiety from the dentist. The usual treatment to alleviate dental anxiety includes dental education, education about the treatment procedure, support, and advice related to dental anxiety.

### 2.4. Procedures

The aim of the psychological intervention was explained to the experimental group. The experimental group received two psychological interventions (psychoeducation and progressive muscular relaxation). During the restorative procedure (i.e., fillings), which was performed by two dentists, participants in the experimental group received another psychological inter-vention (music distraction). The dentist who provided the re-storative treatment to the experimental group participants was aware that the patients had received the psychoeducation and progressive muscular relaxation from his/her colleague. Con-trol group participants received dental treatment as usual from two dentists who were not aware that the patients were control group participants, but who were aware that the patients were involved in the study.

### 2.5. Statistical analyses

Data were entered into SPSS for statistical analysis. De-scriptive statistics were used to calculate the prevalence of dental anxiety. Because the study data were not normally dis-tributed, nonparametric tests were used for analysis. The Mann-Whitney test was used to determine intergroup mean rank differences between pretreatment, posttreatment, and fol-low-up treatment anxiety levels. The Kruskal-Wallis test was used to determine intragroup differences between pretreatment, posttreatment, and follow-up treatment anxiety levels. Dunn’s nonparametric post-hoc test was used for post-hoc analysis.

## 3. Results

### 3.1. Prevalence of dental anxiety

Over 3 months, 64 potential participants were included, of which 33 patients (51.6%) experienced dental anxiety. Out of these 33 patients, two were excluded as they did not meet the inclusion criteria. Demographic characteristics of the study participants are presented in Table 1 No significant differences were found between the experimental and control groups for gender, age, and educational level, indicating that both groups were statistically similar.

**Table 1:** Demographics of experimental and control group subjects.

		Experimental	Control			*P*-value
		N	%	N	%	
Total		15	48.4	16	51.6	-
Gender	Male	4	26.7	8	50.0	0.60
	Female	11	73.3	8	50.0	
Age	≤24	5	33.3	4	25.0	0.72
	25-39	1	6.7	3	18.8	
	40-64	9	60.0	7	43.8	
	≥65	0	0.0	2	12.5	
Educa-tional level	Primary school	1	6.7	3	18.8	0.39
	Secondary school	4	26.7	6	37.5	
	Pre-univer-sity	1	6.7	0	0.0	
	Higher ed-ucation	9	60.0	7	43.8	

*P* ≤ 0.05 was considered statistically significant.

### 3.2. Dental anxiety scores (DAS-R)

No significant differences between the experimental and control group were found in the pretreatment, posttreatment, and follow-up treatment level of dental anxiety Table 2.

**Table 2:** Mean rank of dental anxiety scores.

	Experimental		Control		U-statistic	*P*-value
	MR	SR	MR	SR		
Pretreatment	16.80	252	15.25	244	108	0.57
Posttreatment	13.53	203	18.31	293	83	0.11
Follow-up treatment	15.27	229	14.71	206	101	0.84

Abbreviations: MR, mean rank; SR, sum of ranks.

Significant differences among pretreatment, posttreatment, and follow-up treatment levels of dental anxiety were found in the experimental group (Kruskal-Wallis *%2 =* 14.82, *P* = 0.001, effect size = 0.33) Table 3. Dunn’s Post hoc tests showed significant differences in dental anxiety levels between pre-treatment and posttreatment levels (P = 0.001, effect size = 0.39) and between pretreatment and follow-up treatment (P = 0.001, effect size = 0.57). The effects size was medium. No significant difference was found in anxiety levels between posttreatment and follow-up treatment in the experimental group (P = 0.62). No significant differences (Kruskal-Wallis X2 = 4.41, *P* = 0.11) were found in dental anxiety between pre-treatment, posttreatment, and follow-up treatment levels in the control group.

**Table 3:** Mean rank scores of dental anxiety of patients in the experimental group.

Time of assessment	N	Mean rank	*P*-value
Pretreatment	15	32.60	
Posttreatment	15	18.20	0.00
Follow-up treatment	15	18.09	

*P* ≤ 0.05 was considered statistically significant.

### 3.3. Amelioration of dental anxiety

Of the 15 patients in the experimental group, 10 patients at posttreatment assessment and 11 patients at follow-up no longer suffered from dental anxiety Table 4. Of the 16 par-ticipants in the control group, six at the posttreatment assess-ment and nine at the follow-up treatment assessment no longer experienced dental anxiety. In total 15 participants were in-cluded in the follow-up assessment, as one participant with-drew.

**Table 4:** Percentage of participants experiencing dental anxiety.

Group	Dental anxiety		No dental anxiety		Total	
	N	%	N	%	N	%
Experimental group					
Pretreatment	15	100.0%	0	0.0%	15	100%
Posttreatment	5	33.3%	10	66.7%	15	100%
Follow-up treatment	4	5.8%	11	73.3%	15	100%
Control group					
Pretreatment	16	100.0%	0	0.0%	16	100%
Posttreatment	10	62.5%	6	37.5%	16	100%
Follow-up treatment	6	40.0%	9	60.0%	15	100%

### 3.4. Dental concern (DCA)

Of the 26 items on the DCA, participants experienced five major concerns: extraction, injection, sound or vibration of the drill, number of appointments, and number of treatments per visit. Significant differences in the experimental group for pre-treatment, posttreatment, and follow-up treatment scores of dental concern were found and were related to injection (Kruskal-Wallis *%2 =* 7.69, *P* = 0.02), extraction (Kruskal-Wallis *x2 =* 8.75, *P* = 0.01), and sound/vibration of the drill (Kruskal-Wallis x2 = 20.76, *P* < 0.001) Table 5. No signifi-cant differences were found between pretreatment, posttreat-ment, and follow-up treatment scores of dental concern in the control group.

**Table 5:** Analysis of the source of dental anxiety in patients in the exper-imental group.

Dental concern	Mean rank			*P*-value
	Pretreatment	Posttreatment	Follow-up	
Extraction	30.13	22.17	0.01
Injection	29.70	21.67	17.63	0.02
Sound/vibration of drill	33.10	22.97	12.93	0.00
Number of appointments	25.03	21.80	22.17	0.73
Number of treatments	23.13	22.27	23.60	0.95

*P* ≤ 0.05 was considered statistically significant.

## 4. Discussion

This study assessed the prevalence of dental anxiety in pa-tients at a Malaysian university clinic and investigated the ef-fects of combined psychological interventions on dental anxi-ety. The first major finding was that more than half (51.6%) the patients experienced dental anxiety. This result was com-parable to the 49.0% dental anxiety reported by United King-dom Adult Dental Health Survey in 1998 [3] but lower than the 95.2% reported in Malaysia by Enny et al.[27] in 2012. The variation in the prevalence of dental anxiety could be due to differential patient recruitment time frame, study popula-tions, and/or instruments used to measure dental anxiety. Cor-roboratively, the prevalence of dental anxiety reported by Enny et al. [27] was based on an adolescent population, which may have resulted in a higher prevalence of dental anxiety because younger patients are generally more anxious than older pa-tients [28,29].

The second major finding was that brief psychological treatments comprising psychoeducation, progressive muscular relaxation, and music distraction were effective in reducing mean dental anxiety levels. Previous studies [13,14] used a single intervention to treat dental anxiety, but their interven-tions spanned more than one session and were not brief. In contrast, we aimed to use a simple and brief psychological intervention that could be easily implemented by participants without any prior psychological training. To our knowledge, this study is the first that employed a brief, single-session psychological intervention to reduce dental anxiety. Our re-sults were consistent with aspects of previous studies that in-cluded cognitive therapy with progressive muscular relaxation [23,30] and music distraction [15] and showed efficacy in re-ducing dental anxiety.

Based on the results, a single intervention may be sufficient to reduce dental anxiety in patients. However, one study re-ported that psychological characteristics of patients, severity of dental anxiety, and treatment modes could affect the interven-tion outcomes for dental anxiety [13]. Accordingly, some pa-tients might benefit from a particular intervention, while others might not [13]. Dailey et al. [31] showed that a patient’s anxi-ety could be reduced by filling out a questionnaire that resulted in the patient knowing that the dentist was aware of the anxiety issues. However, these less complex methods may be inade-quate for patients with moderate-to-severe dental anxiety, which may require more comprehensive treatments [32]. Therefore, we combined 3 brief psychological interventions that could be easily applied to increase the probability of suc-cess in reducing dental anxiety in more people.

For example, a patient with dental anxiety exhibits physio-logical symptoms (sweating, palpitation, etc.) and psychologi-cal symptoms (anxiety, apprehension, etc.) [33]. Providing muscular relaxation therapy and distraction techniques alone are not sufficient to reduce the physical and psychological re-actions to dental anxiety because the patient requires psycho-logical education to understand the relationship between dental anxiety and its treatment and progression of dental illness. Second, psychological education is useful for the patient to understand the reactions and consequences of dental anxiety. Therefore, based on the results of the present study, it is rec-ommended that dental practitioners use psychoeducation fol-lowed by relaxation therapy and distraction.

Results of the present study supported our hypothesis that patients who received the psychological interventions would show reductions in posttreatment and follow-up treatment scores of dental anxiety compared to their pretreatment scores. Furthermore, the combined psychological interventions re-mained effective until the follow-up treatment. This result un-derscores the benefits of psychological treatments over phar-macological treatments (intravenous conscious sedation, gen-eral anesthesia, benzodiazepines, and nitrous oxide) [16,23]. Although these medications have been proven to be cost-effective, short-term solutions, they seem to lack long-term benefits, and there is a greater rate of relapse [16,34]. The progressive muscular relaxation technique used in the present study could be adopted by patients as a self-help treatment, thus becoming a lifelong skill for coping with the stresses and strains of daily life in addition to dental anxiety.

On the other hand, the present study found no significant differences between experimental and control groups, which may be due to an adequate amount of advice, support, and dental education concerning treatment and anxiety provided by the dentists to the control group patients. The patients in the experimental group seemed to benefit more (N = 11; 73.3%) from dental anxiety management compared to the control group patients (N = 9; 60%) at follow-up. Although both the psychological intervention and dentist’s advice and education were effective, some participants were unable to overcome their dental anxiety, possibly because of factors not related to dental anxiety or its management [35].

Similarly, participants who received the psychological in-tervention had significant reductions in their anxiety related to extraction, injection, and sound/vibration of the drill. These results supported the effectiveness of a combined psychologi-cal intervention for reducing the common triggers of dental anxiety (injection, sound/vibration of drill). For example, par-ticipants were less anxious about the sound/vibration of the drill because they were listening to music that masked the fear-generating noises.

### 4.1. Practical implications

Many young dentists are less likely to receive adequate training to screen and to treat dental anxiety [36]. Furthermore, choosing a long-term psychological treatment may hamper patient compliance. Based on our results, we recommend that dentists and dental students use a brief psychological interven-tion, which is simple and effective, to reduce dental anxiety. Further, these brief psychological techniques should be incor-porated into the dental curriculum because it is essential for dental students to have adequate insight about dental anxiety and its consequences for treatment. These psychological tech-niques can also be used in public dental hospitals where dental practitioners may not have sufficient time to provide long-term psychological counseling to reduce dental anxiety. In our ex-perience participants were interested in learning the psycho-logical techniques and were willing to use them in the future because they were easy. One of the participants reported that he used these techniques to manage his daily stressors. Anoth-er practical benefit of reducing dental anxiety may be im-proved patient follow-up; all participants of the experimental group came for their follow-up appointments without being reminded.

### 4.2. Study limitations

The present study had several limitations. First, the cohort size was relatively small, accounting for a non-normal distri-bution of the data. Due to small sample size this study could not find dental anxiety differences in gender, age and educa-tional status. There were infrastructural constraints, as a result of which researchers were only able to collect data for three months. Additionally, our inclusion and exclusion criteria re-strained the number of eligible participants. Second, the study design may have introduced experimenter bias. This is because a single-blind procedure was used. Non-significant results found for posttreatment and follow-up treatment may emanate from the length of the study protocol. The posttreatment as-sessment was completed immediately after treatment, and the follow-up treatment assessment was completed two weeks later. A longer follow-up assessment period that followed more than one intervention session may have yielded different ef-fects.

### 4.3. Recommendations for future research

Future studies should consider using a larger sample size to study the effectiveness of the psychological interventions. In addition, using a double-blind study design may preclude ex-perimenter bias. The effectiveness of this brief psychological intervention should be investigated in other populations, such as in adolescents, since that population seems to have higher incidences of dental anxiety. Finally, this psychological inter-vention should be evaluated in patients attending university dental clinic and public dental hospital, as there may be dif-ferences between these two populations in terms of dental anxiety.

## 5. Conclusions

In summary, the combined psychological interventions ef-fectively reduced dental anxiety. Dental practitioners do not need psychological certification to use these techniques, and only simple training is required to employ them.
